# Investigation of Thyroid Disorders in Women with Diabetes in the United Arab Emirates: A Retrospective Cross-Sectional Study

**DOI:** 10.1089/whr.2024.0136

**Published:** 2025-02-17

**Authors:** Bashair M. Mussa, Narjes Saheb Sharif-Askari, Nabil Sulaiman, Salah Abusnana

**Affiliations:** ^1^ Basic Medical Sciences Department, College of Medicine, University of Sharjah, Sharjah, United Arab Emirates.; ^2^ Clinical Sciences Department, College of Medicine, University of Sharjah, Sharjah, United Arab Emirates.; ^3^ Family Medicine and Behavioral Sciences, College of Medicine, University of Sharjah, Sharjah, United Arab Emirates.; ^4^ University Hospital Sharjah, Diabetes and Endocrinology Department, Clinical Science Department, College of Medicine, University of Sharjah, Sharjah, United Arab Emirates.

**Keywords:** thyroid disorders, diabetes mellitus, hypothyroidism, hypertension, UAE women, thiazolidinediones, pioglitazone, GLP-1 analogues, liraglutide

## Abstract

**Aim:**

The present study aims to investigate thyroid disorders (TDs) in women with diabetes mellitus (DM) and the correlation, if any, between TDs and development of hypertension in this group of patients.

**Methods:**

The present study is a retrospective cross-sectional study that was conducted in the United Arab Emirates. Women with DM were randomly selected from the electronic medical records database, and 429 patients were included in the study. The investigation included age, diabetes duration, body mass index, blood pressure, hemoglobin A1c, fasting and random glucose, lipid profile, thyroid function test, and levels of thyroid-stimulating hormone. In addition, the antidiabetic medications used by patients with DM were analyzed.

**Results:**

The majority of the studied population (90%, n = 386) had type 2 DM and 33% (n = 142) had TDs; 42% participants with thyroid dysfunction had hypertension compared with 57% participants with normal thyroid function (odds ratio 0.57; 95% confidence interval 0.33–0.97; *p* = 0.039). It was also found that a smaller number of patients with DM who use pioglitazone as a main antidiabetic medication had thyroid dysfunction (1.4%), whereas participants who used liraglutide were more vulnerable to develop TDs (16.9%).

**Conclusions:**

Two-thirds of women with DM and TDs had hypothyroidism; 42% of women with DM and TDs had hypertension. Liraglutide was seen more in patients with TDs compared with pioglitazone suggesting a potential correlation between TDs and the use of Glucagon-Like Peptide (GLP-1) analogues.

## Background

The pancreas and thyroid gland are key players in the regulation of several endocrine processes, and it is speculated that shared pathophysiological mechanisms lead to the development of diabetes mellitus (DM) and thyroid dysfunction.^1^ However, this association is poorly studied, and many factors that are involved in the pathogenesis of these two disorders are yet to be revealed.^2^ Previously, it was believed that thyroid disorders (TDs) are more related to type 1 DM (T1DM); however, new evidence has emerged to support a correlation between TDs and type 2 DM (T2DM) as well.^3^

The frequency of thyroid dysfunction in patients with DM is much higher compared with the general population based on screening of a randomly selected group of 1310 adult patients with DM.^4^ Twenty-five years later, the Fremantle Diabetes Study Phase II showed similar findings to further support the hypothesis that thyroid abnormalities are more common in patients with DM compared with healthy subjects regardless of the type of DM.^5^ In addition, it has been found that there is no significant difference between the prevalence or incidence of thyroid dysfunction in different types of DM.^5^ More importantly, previous reports have highlighted that female patients had a higher risk of developing thyroid disease compared with males with a prevalence of 12.3%.^4,6^

The diagnosed TDs reported in the DM population were subclinical hypothyroidism, hypothyroidism, hyperthyroidism, and subclinical hyperthyroidism with prevalence of 4.8%–5.1%, 0.9%–1.1%, 0.2%–0.5%, and 0.1%–0.5%, respectively.^4,5^

Possible pathophysiological mechanisms of hypothyroidism in DM include immune cell infiltration into pancreatic islets, decreased expression of glucokinase-specific activity, and GLUT2 protein, which are crucial for the glucose sensing process and oxidative stress, which directly causes pancreas injuries.^7–9^ On the contrary, inconsistent reports have suggested that high levels of thyroxine, which is the main clinical feature of hyperthyroidism, lead to β cell apoptosis by showing an increase in transferase-mediated deoxyuridine triphosphate-biotin nick end labeling and caspase-3 expressions in β cells.^10^ In addition, this TD was linked to impaired glucose-stimulated insulin secretion and decreased half-life of circulating insulin due to decreased sensitivity of adenosine triphosphate-sensitive K^+^ and L-type Ca^2+^ channels of the β cells.^11^

The relationship between TDs and cardiovascular disorders has received more attention recently, and investigators postulated different links between these two disorders. However, these links are still a matter of debate as they highlight ongoing controversy in the outcomes of various studies. It is well documented that DM is a major cardiovascular risk factor and TDs are associated with various cardiovascular adverse outcomes.^12^ Therefore, the association between TDs and DM aggravates the cardiovascular risk, and more studies are warranted to further understand the relationship between TDs and cardiovascular parameters in DM.^12^ A case–control study with 240 subjects with hypothyroidism has shown that there is no correlation between TDs, hypertension, and DM.^12^ This was further confirmed by measurements of blood pressure and plasma fasting glucose.^12^ Another study that was focused on subclinical hyperthyroidism and hypertension also failed to document any association between the blood pressure changes and TDs.^13^ On the contrary, a blood pressure monitoring study in 100 individuals with a new diagnosis of hypothyroidism suggested that systolic and diastolic blood pressures were significantly higher in patients with TDs compared with control subjects.^14^ This variation in the outcomes of the previous studies could be due to different reasons including the small sample size and the heterogenous population that was examined.

Considering the contradiction in these aspects, the present study was designed to include more participants and to focus on a homogenous population of the United Arab Emirates (UAE) with confirmed diagnoses of TDs and DM to investigate the relationship, if any, between TDs and development of hypertension. In addition, antidiabetic medications were also included in the analysis.

## Methods

### Data source

The present study is a retrospective cross-sectional study that was conducted in the University Hospital Sharjah (UHS, Sharjah, UAE). Medical records of female patients with DM were randomly selected from the electronic medical records database by using the list of records in each visit for the diabetes clinic; in each page of the medical records, every second record was considered. All these medical records were created, monitored, and updated by consultant diabetologists and endocrinologists who confirmed the diagnosis of DM and TDs, respectively. Ethical approval for this study was obtained from Ministry of Health and Prevention (MOHAP/DXB/SUBC/NO.14/2017). Consent forms were waived as the study is based on data that were extracted from electronic medical records and did not contain any interventional protocols.

### Study population

**FIG. 1.**
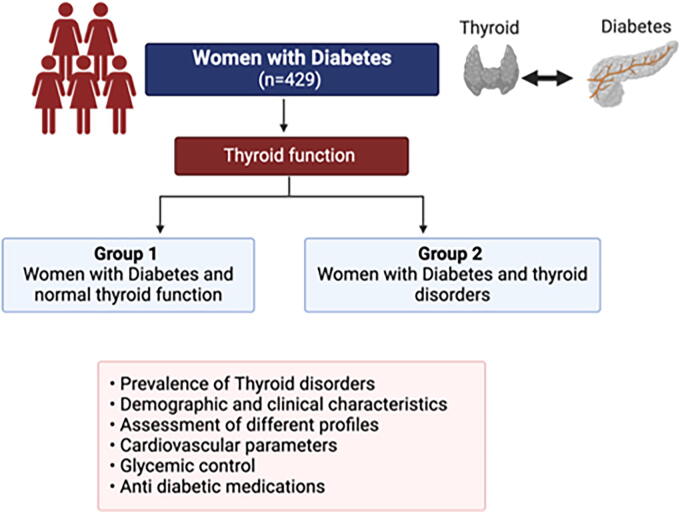
Schematic flow chart showing the groups of the studied population and investigatory profiles.

All the subjects of the study are adult Emirati women with DM who are attending the UHS diabetes clinic quarterly, having initial visit, and having three follow-up visits per annum. A review of the medical records has shown that only 429 medical records have detailed diabetes profiles including diabetes duration (years), age at diagnosis (years), type of diabetes, and diabetes complications and complete thyroid function assessment (Fig. 1).

### Investigation

The following variables were included in the investigation: age (years), diabetes duration (years), body mass index (BMI, kilogram per square meter), systolic blood pressure (sBP, millimeters of mercury), diastolic blood pressure (dBP, millimeters of mercury), hemoglobin A1c (HbA1c, %), fasting and random glucose (millimoles per liter), total cholesterol (millimoles per liter), triglycerides (millimoles per liter), high-density lipoprotein (HDL, millimoles per liter), low-density lipoprotein (LDL, millimoles per liter), thyroid function test, and levels of thyroid-stimulating hormone (milli-international units per liter).

As shown in Figure 1, the studied population was divided into two main groups: (1) Group 1—women with DM and normal thyroid function and (2) Group 2—women with DM and TDs. Several comparisons were conducted to investigate the significant differences between the two groups and to identify correlations, if any, between TDs and cardiovascular parameters. TDs included any abnormalities in thyroid function, either hyperthyroidism or hypothyroidism.

### Statistical analysis

Categorical variables were presented as counts and percentages, while continuous variables were presented as the mean ± standard deviation (SD) or median and interquartile range (IQR), if their distribution was skewed. In the univariate analysis, factors were compared across groups using the χ^2^ test for categorical data and Student’s *t*‐test or Wilcoxon test for continuous data, if their distribution was skewed.^15^ Independent variables of hypothyroidism were identified using a logistic regression model, which was adjusted for age and any other variables that on univariate analysis were significant at *p* < 0.05. All analyses were two‐sided, with a *p*‐value of <0.05 considered statistically significant. The statistical software package used was SPSS 25.00 (SPSS Inc., Chicago, IL, USA).

## Results

### Clinical characteristics of the studied population

The studied population included 429 women with DM, the mean age was 56.5 ± 12.9 years, and BMI was 32.4 ± 6.8 kg/m^2^ (Table 1). The majority had T2DM (90%, n = 386), and the median (IQR) diabetes duration of the women with DM was 11 years (13) and the median (IQR) fasting and random glucose were 7 mmol/L (3.2) and 8.6 mmol/L (5.9), respectively. The control of DM in this group of patients was not optimal as the percentage of HbA1c was 7.4 ± 1.5%. In addition, the assessment of the blood pressure and lipid profile has shown that the median (IQR) of sBP, dBP, HDL, LDL, total cholesterol, and triglyceride were 129 mmHg (24), 75 mmHg (15), 1.4 mmol/L (0.45), 2.6 mmol/L (1.2), 4.5 mmol/L (1.4), and 1.25 mmol/L (0.7), respectively (Table 1).

**Table 1.** Demographic and Clinical Characteristics of the Studied Population

Variables	Total
Subjects (*n*), gender	429, females
Age (years), mean ±SD	56.5 ± 12.9
BMI (kg/m^2^), mean ±SD	32.4 ± 6.8
Diabetes duration (years), median (IQR)	11 (13)
^a^Diabetes type 1/type 2 (%)	10/90
Fasting glucose (mmol/L), median (IQR)	7 (3.2)
Random Glucose (mmol/L), median (IQR)	8.6 (5.9)
HbA1c (%), means ±SD	7.4 ± 1.5
Systolic BP (mmHg), median (IQR)	129 (24)
Diastolic BP (mmHg), median (IQR)	75 (15)
HDL (mmol/L), median (IQR)	1.4 (0.45)
LDL (mmol/L), median (IQR)	2.6 (1.2)
Total cholesterol (mmol/L), median (IQR)	4.5 (1.4)
Triglycerides (mmol/L), median (IQR)	1.25 (0.7)
Thyroid function	
Thyroid disorder, *n* (%)	142 (33)
No thyroid disorder, *n* (%)	287 (67)
TSH, median (mIU/L) (IQR)	1.8 (1.6)
Hypertension, *n* (%)	224 (52.2)

^a^ The percentage of diabetes types was estimated based on the available data on the medical records and/or consultant notes.

BMI, body mass index (calculated as weight in kilograms divided by height in meters squared); BP, blood pressure; HDL, high-density lipoprotein; IQR, interquartile range; LDL, low-density lipoprotein; SD, standard deviation; TSH, thyroid-stimulating hormone.

Investigation of the thyroid function using the complete profiles of 429 medical records has revealed that 33% (142 patients with DM) of the studied population had TDs with the dominance of hypothyroidism as the main pathological feature (two-thirds of the patients with TDs had hypothyroidism, n = 95). On the contrary, 67% (287 patients with DM) of the studied population had normal thyroid function with median (IQR) TSH levels of 1.8 mIU/L (1.6).

### Analysis of metabolic parameters

As shown in Table 2, univariate analysis has revealed that women with diabetes and hypothyroidism were on average 5 years younger than patients without hypothyroidism: 53 ± 15 years versus 58 ± 12 years (*p* < 0.001). In addition, Hb1Ac level was lower in women with diabetes and hypothyroidism compared with those with normal thyroid function; 7.2 ± 1.6% versus 7.5 ± 1.5% (*p* < 0.05). Furthermore, the median (IQR) diabetes duration was 5 years (18) and 15 years (13) in diabetic women with and without hypothyroidism, respectively (*p* < 0.05).

**Table 2.** Univariate Factors of Thyroid Disorders Among UAE Women with DM

Variables	Normal thyroid function (*n* = 287)	Thyroid disorder (*n* = 142)	*p*-Value
Age (years), means ± SD	58 ± 12	53 ± 15	**<0.001**
BMI (kg/m^2^), means ±SD	32.3 ± 6.8	32.43 ± 6.8	0.941
Obese (BMI >25), *n* (%)	246 (86)	120 (85)	0.740
HbA1c (%) means ± SD	7.5 ± 1.5	7.2 ± 1.6	**0.023**
Diabetes duration (years), median (IQR)	15 (13)	5 (18)	**0.040**
Fasting glucose (mmol/L) median (IQR)	7 (3)	7.1 (3.6)	0.700
Random Glucose (mmol/L) median (IQR)	9.8 (5.6)	6.7 (6.3)	0.187
HDL (mmol/L) median (IQR)	1.4 (0.5)	1.3 (0.5)	0.525
LDL (mmol/L) median (IQR)	2.6 (1.2)	2.8 (1.4)	0.094
Total cholesterol (mmol/L), median (IQR)	4.5 (1.5)	4.7 (1.4)	0.121
Triglycerides (mmol/L), median (IQR)	1.2 (0.7)	1.3 (0.7)	0.845
TSH (mIU/L), median (IQR)	1.7 (1.4)	1.8 (2)	0.738
Hypertension, *n* (%)	165 (57)	59 (42)	**0.002**
Systolic BP (mmHg), median (IQR)	131 (25)	125 (25)	**<0.001**
Diastolic BP (mmHg), median (IQR)	76 (14.7)	74 (15)	0.258

Bold values denote statistical significance at the *p* < 0.05 level.

BMI, body mass index (calculated as weight in kilograms divided by height in metres squared); BP, blood pressure; HDL, high density lipoprotein; IQR, interquartile range; LDL, low density lipoprotein; SD, standard deviation; TSH, thyroid stimulating hormone.

As shown in Table 2, hypertension was significantly higher in patients with DM with normal thyroid function compared with those with TDs. Similarly, sBP was lower in the latter compared with the patients with systolic blood pressure with normal thyroid function.

The median (IQR) sBP was significantly lower in women with DM with hypothyroidism compared with those without hypothyroidism; 125 mmHg (25) versus 131 mmHg (25)  (*p* < 0.001). In addition, hypertension was less frequent in women with hypothyroidism than in those without this disorder (42% vs. 57%; *p* = 0.002).

Multivariate analysis has shown that patients with DM with TDs were less likely to have hypertension (odds ratio 0.57; 95% confidence interval 0.33–0.97; *p* = 0.039). On the contrary, no association was observed between hypothyroidism and other variables including age, diabetes duration, and HbA1c (Table 3).

**Table 3.** Multivariate Factors of Thyroid Disorders Among Women with Diabetes

Variables	Adjusted OR (95% CI)	*p*-Value
Age (years), means ± SD	0.99 (0.97–1.013)	0.405
HbA1c (%)	0.89 (0.73–1.09)	0.253
Hypertension, *n* (%)	0.57 (0.33–0.97)	**0.039**
Diabetes duration (years)	1 (0.99–1.01)	0.505

Bold values denote statistical significance at the *p* < 0.05 level.

Independent variables of hypothyroidism were identified using a logistic regression model, which was adjusted for age (years), HbA1c (%), diabetes duration (years), and systolic BP (mmHg).

CI, confidence interval; HbA1c, hemoglobin A1c; OR, odds ratio.

### Analysis of antidiabetic medications

**FIG. 2.**
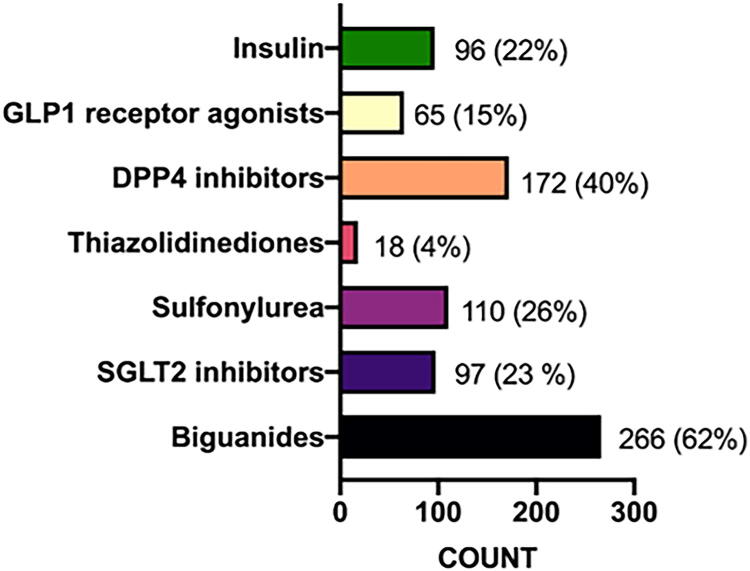
Percentage of the prescribed pharmacological treatment for diabetes.

Further analysis of the medications for the patients with TDs (n = 142 patients) has demonstrated that 94 of 95 patients with hypothyroidism were on levothyroxine medication. On the contrary, antidiabetic agents included biguanides (266, 62%), Dipeptidyl peptidase-4 (DPP4) inhibitors (172, 40%), sulfonylurea (110, 26%), Sodium-Glucose Transport Protein 2 (SGLT2) inhibitors (97, 23%), insulin (96, 22%), Glucagon-like peptide-1 (GLP1) receptor agonists (65, 15%), and thiazolidinediones (18, 4%) (Fig. 2).

The outcomes of the present study have shown that a smaller number of patients with TDs used thiazolidinediones (pioglitazone; 1.4% vs. 5.6%). On the contrary, more patients with TDs used GLP1 receptor agonists (liraglutide; 16.9% vs. 9.4%), and these findings were statistically significant (Table 4).

**Table 4.** Prescribed Pharmacological Treatment for Diabetes in Normal Thyroid Function Versus Thyroid Disorders (*n* = 429)

Variables	Normal thyroid function (*n* = 287)	Thyroid disorder (*n* = 142)	*p*-Value
Number of medications, median (IQR)	2 (2)	2 (2)	0.489
Biguanides, metformin, *n* (%)	179 (62.6)	87 (61.3)	0.833
SGLT2 inhibitors	68 (23.8)	29 (20.4)	0.435
Empagliflozin, *n* (%)	16 (5.6)	7 (4.9)	0.999
Dapagliflozin, *n* (%)	52 (18.2)	23 (16.2)	0.686
Sulfonylurea, *n* (%)	80 (28)	30 (21.1)	0.158
Glimepiride, *n* (%)	5 (1.7)	1 (0.7)	0.668
Gliclazide, *n* (%)	75 (26.2)	29 (20.4)	0.231
Thiazolidinediones, pioglitazone, *n* (%)	16 (5.6)	2 (1.4)	**0.043**
DPP4 inhibitors, *n* (%)	116 (40.6)	54 (39.4)	0.835
Lingaliptin, *n* (%)	10 (3.5)	6 (4.2)	0.788
Sitagliptin, *n* (%)	59 (20.6)	28 (19.7)	0.899
Vildagliptin, *n* (%)	47 (16.4)	22 (15.5)	0.899
GLP1 receptor agonists, *n* (%)	37 (12.9)	28 (19.7)	0.085
Liraglutide, *n* (%)	27 (9.4)	24 (16.9)	**0.027**
Dulaglutide, *n* (%)	10 (3.5)	4 (2.8)	0.999
Insulin, *n* (%)	67 (23.4)	29 (20.4)	0.539

Bold values denote statistical significance at the *p* < 0.05 level.

## Discussion

It is well documented that DM and thyroid dysfunction are closely related regardless of the type of DM.^16^ Untreated TDs produce a negative impact on the metabolic control of patients with DM, and therefore, it is crucial to investigate the outcomes of this association, particularly in females who are at higher risk to develop these two disorders, simultaneously.^4,6^

Unexpectedly, the findings of the present study have shown that female patients with thyroid dysfunction have significantly lower HbA1c levels compared with those with normal thyroid function. It is noteworthy that the low levels of HbA1c in thyroid dysfunction were observed in the univariate analysis; however, when the age and diabetes duration were adjusted in the multivariate analysis, no more significant differences in the HbA1c were reported.

Previous reports have demonstrated the involvement of several factors in the modulation of HbA1c levels including red blood cell turnover.^17^ Interestingly, it was found that HbA1c levels may change due to altered thyroid status, and this was observed in patients with DM and healthy participants.^17^^,^^18^ It is known that thyroid hormones stimulate erythrocyte production, and any reduction in their levels as in hypothyroidism leads to hypoproliferative erythropoiesis.^19^ Therefore, abnormality in HbA1c levels in hypothyroidism does not accurately reflect glycemic status nor control.^18^

Another significant finding of the present study is that diabetes duration in females with DM with thyroid dysfunction was shorter compared with the patients with DM with normal thyroid function. Although previous clinical studies reported a linear positive association between diabetes duration and thyroid dysfunction in more than 5 years, other basic studies have demonstrated that in experimental long-term DM, thyroid hormones were returned to normal range, suggesting the development of adapting processes at the thyroid level.^20,21^

One of the main findings of the present study is that the women with DM with thyroid dysfunction were less likely to develop hypertension compared with women with normal thyroid function. Although numerous studies have investigated the relationship between thyroid dysfunction and hypertension, none of these studies focused on female patients with DM, and this highlights the novelty of the present study.^12–14^^,^^22^ A strong relationship between thyroid function and blood pressure has been established based on the direct and indirect influence of the thyroid hormones on cardiovascular system. This includes manipulation of systemic vascular resistance, resting heart rate, left ventricular contractility, and blood volume by these hormones.^23^^,^^24^ In addition, endothelial function is closely related to the thyroid function given that thyroid hormones enhance the activation of endothelial Nitric Oxide (NO) synthase through the Phosphatidylinositol 3-Kinase/Protein Kinase B (PI3K/Akt) pathway and the renin–angiotensin–aldosterone system and, furthermore, positively control cardiac myocyte β1‐adrenergic receptors in animal models.^25–28^ In addition, results from ambulatory blood pressure monitoring studies have shown that sBP and dBP were significantly higher in patients with hypothyroidism compared with healthy volunteers.^14^ However, other studies in adults with euthyroid failed to establish a relationship between the thyroid hormones and elevated blood pressure.^29^ Although cardiovascular complications have been associated with both hypothyroidism and hyperthyroidism, increased levels of thyroid hormones seemed to be associated with hypertension.^30^ Given that hypothyroidism is the main pathological feature of our present studied population, it is reasonable to suggest that this feature may represent a risk factor for development of hypertension. This suggestion is further supported by echocardiography studies, which have shown that pulmonary hypertension is the most common complication in patients with hyperthyroid and high cardiovascular mortality was observed among patients with hyperthyroidism.^30,31^

On the contrary, the correlation between cardiovascular abnormalities and hypothyroidism is still debated, and it seems that other factors including hyperlipidemia influence this correlation.^32^ In line with these findings, other clinical reports have revealed a positive association between thyroid hormones and triglyceride levels and reduced endothelium-dependent vasodilation.^33^ This may provide a plausible explanation for our present outcomes, which showed that 42% of women with DM and thyroid dysfunction have developed hypertension.

Analysis of pharmacological treatment of hypothyroidism and DM has shown that levothyroxine was the main therapeutic agent for the former, and there were seven subclasses to treat the latter with the dominance of biguanides. Further analysis has been conducted to evaluate the use of antidiabetic medications in patients with normal thyroid function compared with those with TDs. The outcomes of the analysis revealed a significant difference in two subclasses of antidiabetic medications: thiazolidinediones (pioglitazone) and Glucagon-like peptide 1 (GLP1) receptor agonists (liraglutide). It was found that a smaller number of patients with DM who use pioglitazone as the main antidiabetic medication had thyroid dysfunction. Pioglitazone is a peroxisome proliferator-activated gamma (PPARγ) agonist that mediates the antidiabetic action via binding to nuclear PPARγ receptor.^34^ It regulates several genes related to glucose and lipid metabolism, which eventually increases insulin sensitivity.^34^ Our results show that most of the patients with DM who use pioglitazone have normal thyroid function, suggesting that this agent may have protective effects against thyroid dysfunction or at least is not associated with the development of any thyroid abnormalities. In agreement with these findings, previous reports have demonstrated beneficial effects on thyroid function, including anti-inflammatory and antitumor effects.^35,36^ Therefore, it has been suggested that pioglitazone can act as a therapeutic agent for some of the thyroid carcinomas.^37^ Given that thyroid hormone receptors belong to the same superfamily of nuclear hormone receptors, it is not surprising to know that there is a significant crosstalk between peroxisome proliferator and thyroid hormone signaling pathways with a synergistic or opposing nature.^37^ A more recent report has shown that pioglitazone has an opposing effect on the levels of thyroid hormones. However, several factors including the sample size and gender of the studied population may explain these outcomes.^38^ It is noteworthy that the design of the present study is cross sectional and the duration of the thyroid dysfunction and the duration of the pioglitazone’s use were not reported. Therefore, it would be challenging to draw a solid conclusion on the effects of pioglitazone on thyroid function solely based on the present study.

On the contrary, the present study has revealed that patients with DM who use liraglutide are more vulnerable to develop TDs. Clinical studies of the effect of GLP1 analogues on thyroid function have demonstrated a significant reduction in thyroid-stimulating hormone with no change in thyroid volume in patients with DM without thyroid disease.^39^

### Limitations and directions for future research

The main limitations of the present study are the small sample size and the retrospective nature of the study design. Follow-up studies should be designed to follow the patients in a prospective longitudinal model to better our understanding of the factors involved in the pathogenesis of TDs in women and men with DM. This study did not evaluate the presence of thyroid dysfunction in men with DM; therefore, it cannot determine whether the observations are applicable to men as well. In addition, it would be of great interest to investigate different types of TDs (hyperthyroidism and hypothyroidism) and different types of diabetes (T1DM and T2DM) in a segregated manner to explore more details about the pathological mechanisms of each type.

## Conclusions

In conclusion, most of the studied population were T2DM with hypothyroidism; 42% of women with DM and TDs had hypertension The analysis of the antidiabetic medications has shown that the use of liraglutide was seen more in patients with TDs compared with pioglitazone.

## Data Availability

The research data used in the preparation of the article are available from B.M.M. upon reasonable request from researchers who have approval to access confidential information.

## Abbreviation Used

BMI: Body Mass Index
CI: Confidence Interval
dBP: Diastolic Blood Pressure
DDP4: Dipeptidyl peptidase-4
DM: Diabetes Mellitus
GLP-1: Glucagon-like peptide 1
HbA1C: Hemoglobin A1c
HDL: High Density Lipoprotein
IQR: Interquartile Range
LDL: Low Density Lipoprotein
NO: Nitric Oxide
OR: Odd Ratio
PI3K/Akt: Phosphatidylinositol 3-Kinase/Protein Kinase B
PPARγ: Peroxisome Proliferator-Activated Gamma
RBCs: Red Blood Cells
sBP: Systolic Blood Pressure
SD: Standard Deviation
SGLT2: Sodium-Glucose Transport Protein 2
T1DM: Type 1 Diabetes
T2DM: Type 2 Diabetes
TDs: Thyroid Disorders
UAE: United Arab Emirates
UHS: University Hospital Sharjah
